# An Optimized Approach for Prostate Image Segmentation Using K-Means Clustering Algorithm with Elbow Method

**DOI:** 10.1155/2021/4553832

**Published:** 2021-11-15

**Authors:** Rachid Sammouda, Ali El-Zaart

**Affiliations:** ^1^Department of Computer Science, College of Computer and Information Sciences, King Saud University, Riyadh, Saudi Arabia; ^2^Department of Mathematics and Computer Science, Faculty of Sciences, Beirut Arab University, Beirut, Lebanon

## Abstract

Prostate cancer disease is one of the common types that cause men's prostate damage all over the world. Prostate-specific membrane antigen (PSMA) expressed by type-II is an extremely attractive style for imaging-based diagnosis of prostate cancer. Clinically, photodynamic therapy (PDT) is used as noninvasive therapy in treatment of several cancers and some other diseases. This paper aims to segment or cluster and analyze pixels of histological and near-infrared (NIR) prostate cancer images acquired by PSMA-targeting PDT low weight molecular agents. Such agents can provide image guidance to resection of the prostate tumors and permit for the subsequent PDT in order to remove remaining or noneradicable cancer cells. The color prostate image segmentation is accomplished using an optimized image segmentation approach. The optimized approach combines the k-means clustering algorithm with elbow method that can give better clustering of pixels through automatically determining the best number of clusters. Clusters' statistics and ratio results of pixels in the segmented images show the applicability of the proposed approach for giving the optimum number of clusters for prostate cancer analysis and diagnosis.

## 1. Introduction

As per the American Cancer Society [1], in 2020, the United States is relied upon to have 191,930 new instances of prostate disease, and the number of deaths due to prostate malignancy will reach 33,330 [2]. Prostate malignant growth has outperformed cellular breakdown in the lungs by ending up being the most widely recognized because of the broad increment of separating 2016 [3]. The number of prostate cancer patients who have been treated is increasing significantly [4]. In 2014, the report issued by Data of Saudi Cancer Registry has exposed that the rank of prostate cancer was the fifth and creates 6.1% of overall cancers in men in the Saudi Arabia [5]. Also, in Saudi Arabia for years between 2001 and 2008, there are 7.7 per 100,000 men, and 5.1 per 100,000 men were estimated as prostate cancer of the age-standardized incidence rate (ASIR) and the age-standardized mortality rate (ASMR), respectively, by the International Agency for Research on Cancer (IARC) [6].

During the last years, physicists are still working to improve imaging techniques, and they continue updating them in a multidimensional space field to aid radiologists in recognition and analysis of cancer cells. Lately, Neuman et al. [7] have exposed improving the prostate cancer surgical treatment and reducing the margins of positive surgical rate by the near-infrared (NIR) fluorescence probe YC-27 in real time laparoscopic extirpative surgery.

One noninvasive therapy is photodynamic therapy (PDT), which is clinically used in cancer treatment and the other diseases [8, 9]. PDT utilizes photosensitizers that become active when they were visible to light in the presence of oxygen. The reactive oxygen species like singlet oxygen that are formed by the active drug kill nearby cells. Many agents that include phthalocyanines and porphyrins have been assessed as photosensitizers [10, 11]. In an effort to improve the accuracy of prostate cancer identification, an optimized light source-based near-infrared (NIR) fluorescence imaging system is used for reducing the positive surgical margins (PSM) of cancer cells [7]. The main PDT treatment challenge is the accumulation of off-target tissue and photosensitizer activation, killing cells in normal tissue [12]. Minimizing the side effects and generating better outcomes therapeutically required the optimal method of a particularly selective delivery for photosensitizers.

PSMA is an exclusive membrane bound glycoprotein, initially exposed in the androgen-dependent prostate adenocarcinoma of the human cell by means of a monoclonal [13]. Therefore, the PSMA is considered as an overexpressed on analysis of prostate cancer. There is a correlation between PSMA expression in cancer tissues and the disease stage and score of Gleason [14]. In cells of prostate cancer taken from hormone-refractory patients, the PSMA expression is also higher [14] and increased to be shown as an independent marker for the disease recurrence [9]. Moreover, the PSMA can be also stated in neovasculature for various solid tumors [15]. Unsupervised approach based on Hopfield neural network classifier is used in [16] for NIR prostate image segmentation.

Precise prostate image segmentation and volume assessment assume a fundamental part in the conclusion and therapy of prostate-related illnesses, particularly the organizing evaluation of prostate malignant growth. As of now, attractive reverberation imaging (MRI) has turned into the principle imaging strategy for helping prostate finding because of its high-goal and delicate tissue contrast [17].

Nevertheless, the assessment of the prostate images depends on the visual investigation accomplished by the radiologist, which is very tedious, time-consuming, subjective, and complicated. Thus, in the previous decade, different prostate segmentation techniques have been proposed. For example, Shi et al. [18] took advantage of the coupled element feature and spatial-constrained to assess the 3D prostate probability map and utilize the multimap fusion approach to produce the last segmentation results.

A deformable segmentation technique for prostate images was proposed by Guo et al. [19] to combine the sparse patch matching technique and deep feature learning method for prostate image segmentation. These strategies have really shown a promising result in the prostate cancer diagnosis throughout the automatic segmentation of prostate images. However, because of the heterogeneity of the prostate gland, the low difference between the gland and neighboring tissues, and the absence of clear boundaries and strong edges [20], the segmentation of prostate images is still remaining as a part of current research studies and a challenging task [21, 22].

Ushinsky et al. [23] introduced a prostate organ segmentation approach using a hybrid U-Net convolutional neural network (CNN). The approach is used for automatic localization and segmentation of prostates from multiparametric MRIs (mpMRIs). The author trained the CNN on 7774 MRI of 287 patients to achieve 0.974 for the Pearson correlation coefficient metric and 0.898 for the mean Dice score. However, further works should be conducted to develop some pattern recognition methods for lesion quantification and localization.

Pan et al. [24] proposed a prostate segmentation model on the 3D magnetic resonance images (MRIs). The model contains two stages: variable input-based uncertainty measures and an uncertainty-guided postprocessing method. The author validated the robustness of the model and showed that the label smoothness has been improved significantly by applying the uncertainty-guided postprocessing method.

In this study, an optimized approach for NIR prostate image segmentation is proposed using the k-means clustering algorithm with the elbow method. The optimized approach can give better clustering of pixels by determining automatically the best number of clusters. The NIR images of prostate are acquired throughout PSMA-1-PC413, two PSMA-1-based PDT conjugates, and PSMA-1-IR700. In clinical trials, currently, the Pc413 is a second generation analogue of phthalocyanine PDT drug Pc4 [25], and commercially, the IR700 is available near-infrared dye that has presented to take PDT events [26].

The core contributions of this research work can be given as follows:

- An optimized approach is proposed for prostate image segmentation using the k-means clustering algorithm with elbow method. The approach is able to find the best number of clusters to k-means for better prostate images segmentation and supporting the radiologist to give accurate diagnosis regarding to the visual inspection of prostate malignant growth.
- The proposed approach is implemented by combining the k-means clustering algorithm with elbow method and evaluating it on two datasets by analyzing the clusters' statistics and ratio results of pixels in the segmented images.

The reminder of the paper is outlined as follows.  Section 2 describes the datasets and research methods used in this study work.  Section 3 presents the experimental results and discussion in more detail.  Section 4 concludes the research work highlighting the future direction of the study.

## 2. Materials and Methods

### 2.1. Datasets

**Figure 1:**
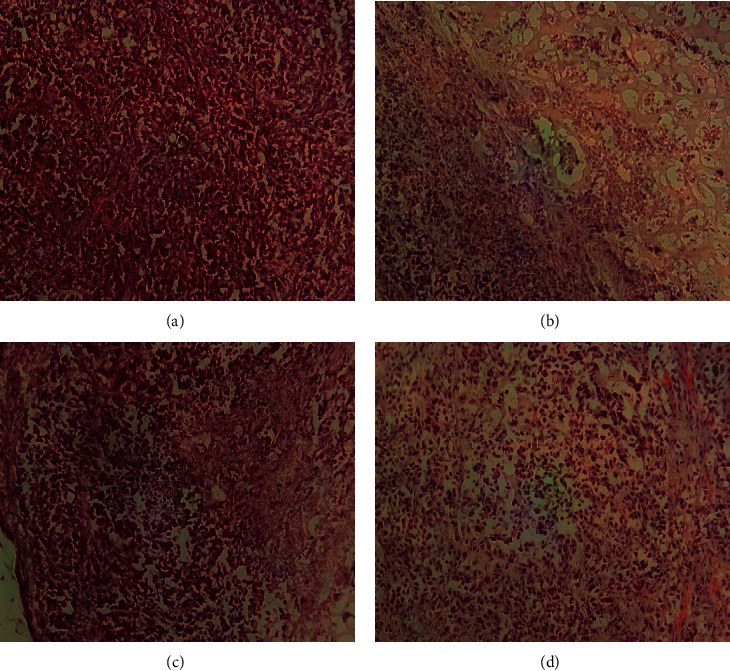
NIR images of dataset 1: (a) 0.5 mg/kg PSMA-1-Pc413 with no light irradiation, (b) 0.5 mg/kg PSMA-1-Pc413 with 150 J/cm2 light irradiation, (c) 0.5 mg/kg PSMA-1-IR700 with no light irradiation, and (d) 0.5 mg/kg PSMA-1-IR700 with 50 J/cm2 light irradiation.

Two datasets are used to validate the applicability and effectiveness of our proposed clustering method. They are collected from two different sources for generating dataset 1 and dataset 2. The dataset 1 is acquired from our previous work [16]. It contains four samples of NIR color images getting PSMA-1-IR700 and PSMA-1-PC413 with and without the light irradiation.  Figure 1 shows the data sample images of dataset 1.

**Figure 2:**
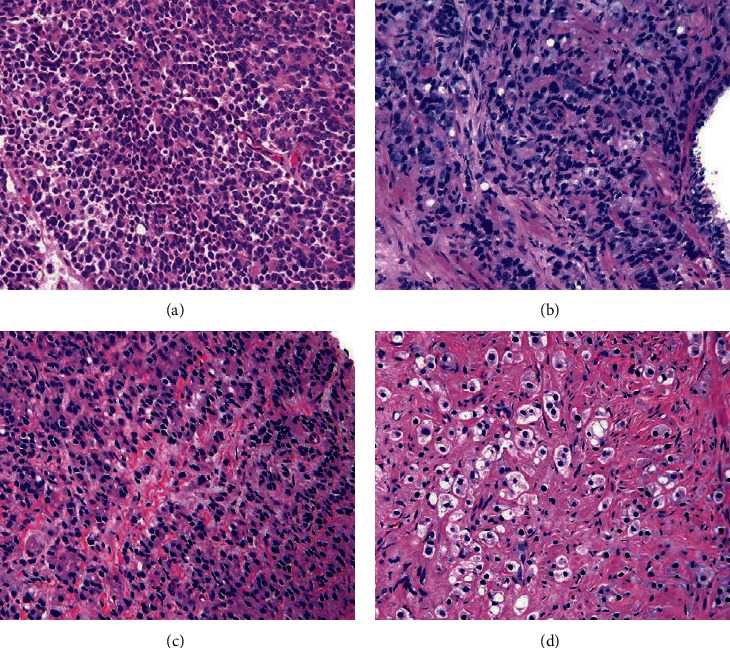
Histological color images of dataset 2: (a) sheets of cancer with rosette formation, (b) small nests and cords of tumor with scattered clear vacuoles, (c) nests and cords of cells with only vague attempt at Lumina formation, and (d) solid nests of cancer.

Additionally, dataset 2 consists of four prostate histological color images obtained from pathology dataset images collected by the Pathology Laboratory of the Johns Hopkins University and available in its website link (https://pathology.jhu.edu/prostatecancer/).  Figure 2 presents the data sample images of dataset 2. Gleason pattern 5 (GP5) is the type of selected images in this dataset. The Gleason system is a useful indicator used to as a predictive outcome value for clinical diagnosis of prostate cancer patients. It has a grade of 1 for the cancerous prostate tissue that looks as a normal tissue and a grade of 5 for the abnormal prostate tissue due to cancer cells and their growth patterns.

### 2.2. Research Methods

In this subsection, we explain the research methods adopted to propose the research work approach. The approach aims to develop an optimized clustering method for analyzing the distribution of pixels in the histological and near-infrared (NIR) prostate cancer images. It is built based on the traditional k-means clustering algorithm and elbow criterion technique described in the following subsections.

#### 2.2.1. K-Means Clustering Algorithm

Clustering methods are algorithms used for grouping the objects or regions into clusters based on their attributes. Each cluster can be defined as a group of instances which are similar between them and dissimilar to the instances, which are in other clusters. In machine learning field, clustering-based methods belong to unsupervised learning concept. The unsupervised learning concept can train from the features of available unlabeled data. In other words, clustering can be defined as a static-classification of similar entities or objects into several different groups or more subsets. Consequently, the member objects in the same group have similar attributes, which is commonly contained in the coordinate system with shorter spatial distance. In prostate cancer image segmentation and analysis, similar pixels are grouped together to form clusters or segments according the similarity condition, which are well-defined between pixels [17]. K-means is one of more common and effective unsupervised algorithms, used for clustering task. It has a good clustering effect and simple to implement. There are some variants of the k-means algorithm. The simple notion behind the k-means algorithm is explained by the following lines. Suppose that a given dataset of data points (*x*_1_, *x*_2_, *x*_3_,…, *x*_*n*_) is divided into *K* number of clusters (*C*_1_, *C*_2_, *C*_3_,…, *C*_*k*_) regarding the distance value between the data points in this dataset. Then, the goal is minimizing the sum of squared errors within cluster(SSEWC), which can be computed as follows:

(1) $$\begin{array}{c} \text{SSEWC}=\underset{C}{\text{argmin}}\sum_{i=1}^{k}\sum_{x\in C_{i}}x-\mu_{i}^{2}=\underset{C}{\text{argmin}}\sum_{i=1}^{k}\left|C_{i}\right|\mathrm{var}\;C_{i}, \end{array}$$

where |*C*_*i*_| represents the size of cluster *i*, var *C*_*i*_ is the variance of cluster *i*, and ***μ***_*i*_ is the mean of data points in *C*_*i*_. This is similar to minimizing the data points pairwise squared deviations of the same cluster as follows:

(2) $$\begin{array}{c} \text{SSEWC}=\underset{C}{\text{argmin}}\sum_{i=1}^{k}\frac{1}{2\left|C_{i}\right|}\sum_{x,y\in C_{i}}x-y^{2}. \end{array}$$

Also, we deduce the difference between data points from their mean using identity as follows:

(3) $$\begin{array}{c} \sum_{x\in C_{i}}x-\mu_{i}^{2}=\sum_{x\neq y\in C_{i}}{\left(x-\mu_{i}\right)}^{T}\left(\mu_{i}-y\right). \end{array}$$

By equivalence and because the total variance is normally constant, minimizing the sum of squared errors within cluster (SSEWC) is equal to maximizing the sum of squared error between clusters (SSEBC) or between data points of different clusters, computed as follows:

(4) $$\begin{array}{c} \text{SSEBC}=\underset{C}{\text{argmax}}\sum_{i=1}^{k}\left|C_{i}\right|\mu -\mu_{i}^{2}, \end{array}$$

where ***μ*** is the mean of clusters, ***μ***_*i*_ is the mean of data points in *C*_*i*_, and |*C*_*i*_| is the size of cluster *i*.

Hence, the *k*-means algorithm aims to make the distance between the clusters of the dataset as large as possible and make the distance between the data points of the same cluster as small as possible.

The main steps of the k-means clustering algorithm can be summarized as follows:

- K centroids are created; then, each point is assigned to the nearest centroid.
- The centroid is recalculated. This process is repeated several times until the result of cluster allocation of data points no longer changes.

The main advantages of k-means include a number of points: first, it belongs to unsupervised learning; no training set is required; second, the principle of k-means is very simple, and it is easier to implement; and finally, the result of k-means is better interpretable. In contrast, one disadvantage of k-means comes in the difficulty of choosing an inappropriate value of *K* that might lead to poor clustering results. This is why it is necessary to perform feature checks to determine the number of clusters in the dataset, and this depends on the application domain. Other disadvantages of the k-means algorithm represented in it might converge to a problem of local minimum; it has a slower convergence on large-scale of data sets; and it is more sensitive to outliers.

#### 2.2.2. Proposed Method

**Figure 3:**
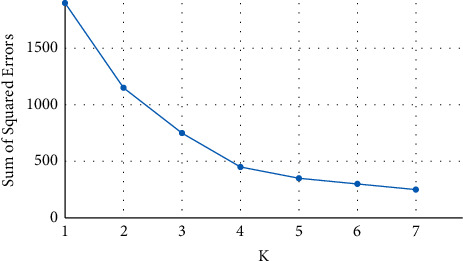
An example of how elbow criterion technique works.

The proposed method aims to segment or cluster and analyze pixels of histological and near-infrared (NIR) prostate cancer images by improving and optimizing the traditional k-means algorithm by finding the number of clusters through elbow criterion technique. Elbow criterion technique is a heuristic method applied to determine the number of clusters of the data points in a dataset. Elbow technique is used to obtain the optimal number of clusters for a set of data points because it is an empirical method, simple and easy to implement. By applying the k-means clustering algorithm, the elbow technique plots the explained variations through the number of clusters and picks the curve of elbow to get the number of clusters. It depends on computing the sum of squared errors within-cluster (SSEWC) given in Equation (1) of all data points to represent the quality of aggregation between data point in the same cluster and separation between clusters. The core idea of the elbow technique is explained as follows:

- As the number of clusters *K* increases, the model separation is more distinguished, and the amount of aggregation for each cluster will gradually be increased; therefore, the SSEWC will gradually and obviously become smaller.
- When *K* is less than the true number of clusters, increasing the value of *K* significantly increases the amount of aggregation for each cluster. SSEWC will be decreased, and once *K* will reach the true number of clusters, the amount of aggregation achieved using *K* will be increased. Therefore, the return will be decreased quickly, and the SSEWC will be decreased sharply. Consequently, it will be flattened out when the *K* value remains increasing. This means that the relationship between SSEWC and *K* can be represented in the elbow shape corresponding to the *K* value, which is the number of true clusters for the example points. For instance, in Figure 3, it obvious that the highest curvature of elbow is at *K* value of 4. Hence, the best number of clusters will be 4.

## 3. Experimental Results and Discussion

**Figure 4:**
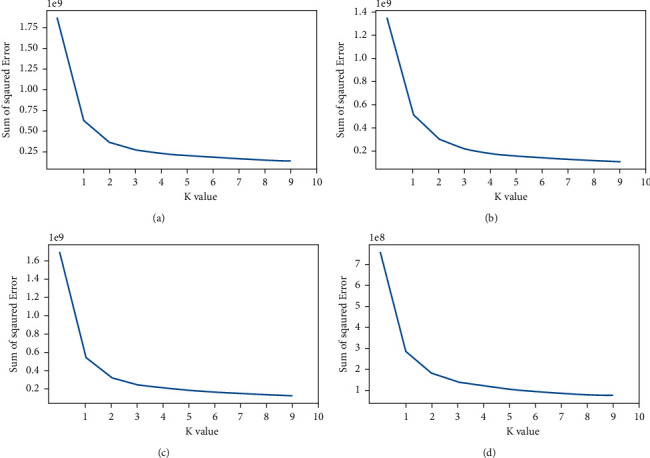
The curve plots of elbow criterion technique for prostate images in dataset 1: (a) curve plot of image 1, (b) curve plot of image 2, (c) curve plot of image 2, and (a) curve plot of image 4.

This section gives and discusses the results of the experiments conducted by the proposed image clustering method on the prostate dataset images described in subsection 2.1. At first, we present the curve plots of elbow criterion technique for prostate images in dataset 1 as displayed in Figure 4. It shows the highest curvature of elbow in all plots at *K* = 4, and after this value, the curvature seems to be stable and does not have much changes. Therefore, the best number of clusters for the images of dataset 1 is 4.

**Figure 5:**
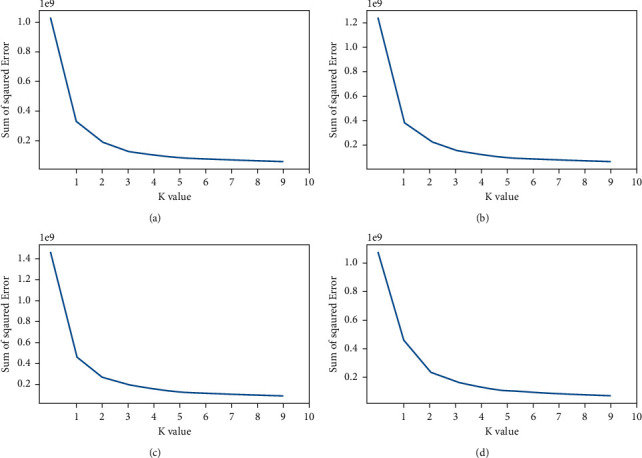
The curve plots of elbow criterion technique for prostate images in dataset 2: (a) curve plot of image 1, (b) curve plot of image 2, (c) curve plot of image 2, and (a) curve plot of image 4.

Similarly, Figure 5 gives the plot curves of elbow criterion technique for the images in dataset 2. From Figure 5, we can see that value 4 is the best number of clusters in which the changes of elbow curvature are not higher than the other number of clusters in all plots, and after this value, the curvature seems to be stable and does not much change.

**Figure 6:**
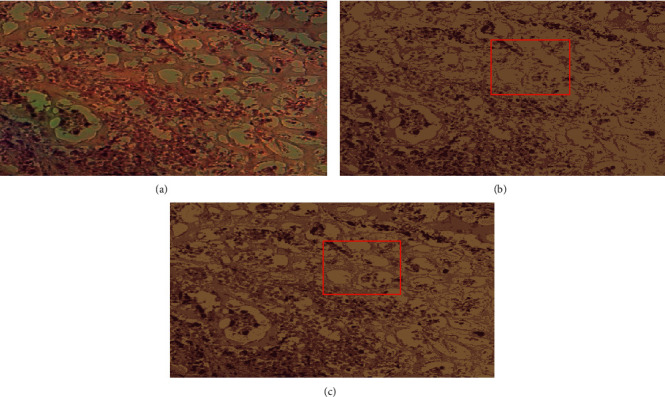
An original image from dataset 1 with their segmented images: (a) the original image, (b) its segmented image with *K* = 3, and (c) its segmented image with *K* = 4.

**Figure 7:**
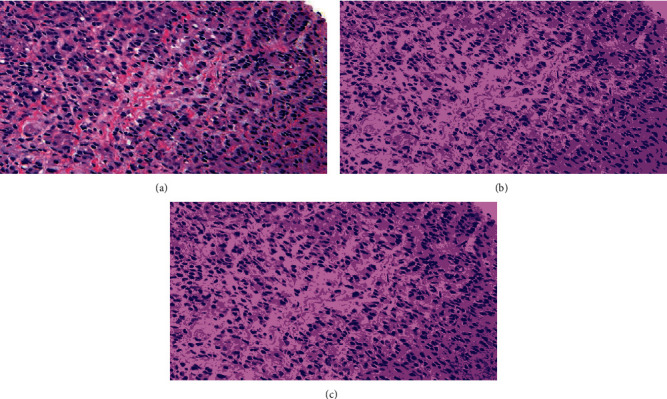
An original image from dataset 2 with their segmented images: (a) the original image, (b) its segmented image with *K* = 3, and (c) its segmented image with *K* = 4.

To show the effect of number of clusters selected by elbow criterion technique, Figures 6(a) and 7(a) show an original image from dataset 1 and an original image from dataset 2 with their segmented images at *K* = 3 and *K* = 4 in Figures 6(b), 7(b), 6(c), and 7(c), respectively. We notice from the red bounding box in Figure 6(b) and Figure 6(c) that the value 4 for the number of clusters is suitable to cluster image pixels into appropriate regions with a good separation and without overlapping between these regions.

**Figure 8:**
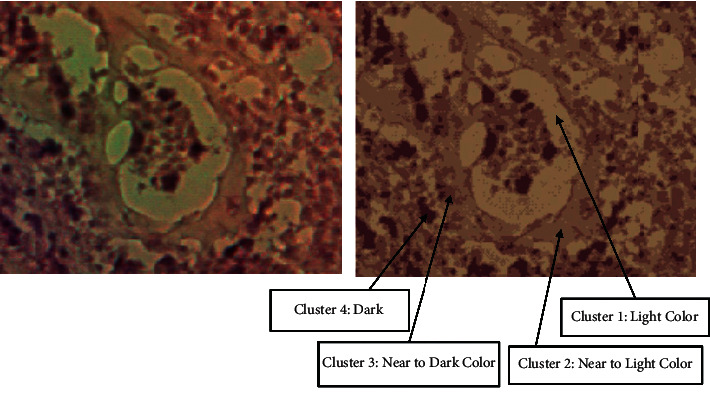
Visualization of clusters' regions with their labels for a part of an image taken from dataset 1.

**Figure 9:**
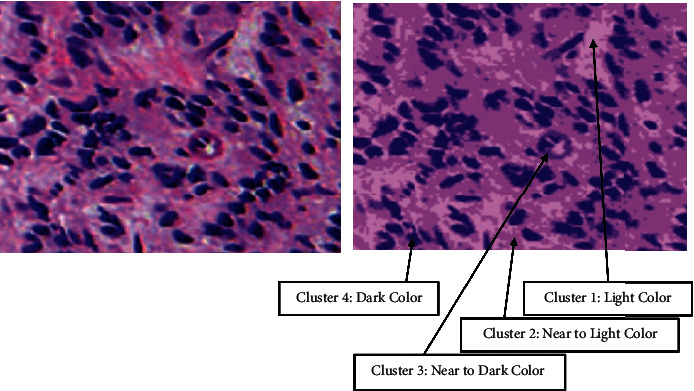
Visualization of clusters' regions with their labels for a part of an image taken from dataset 2.

**Figure 10:**
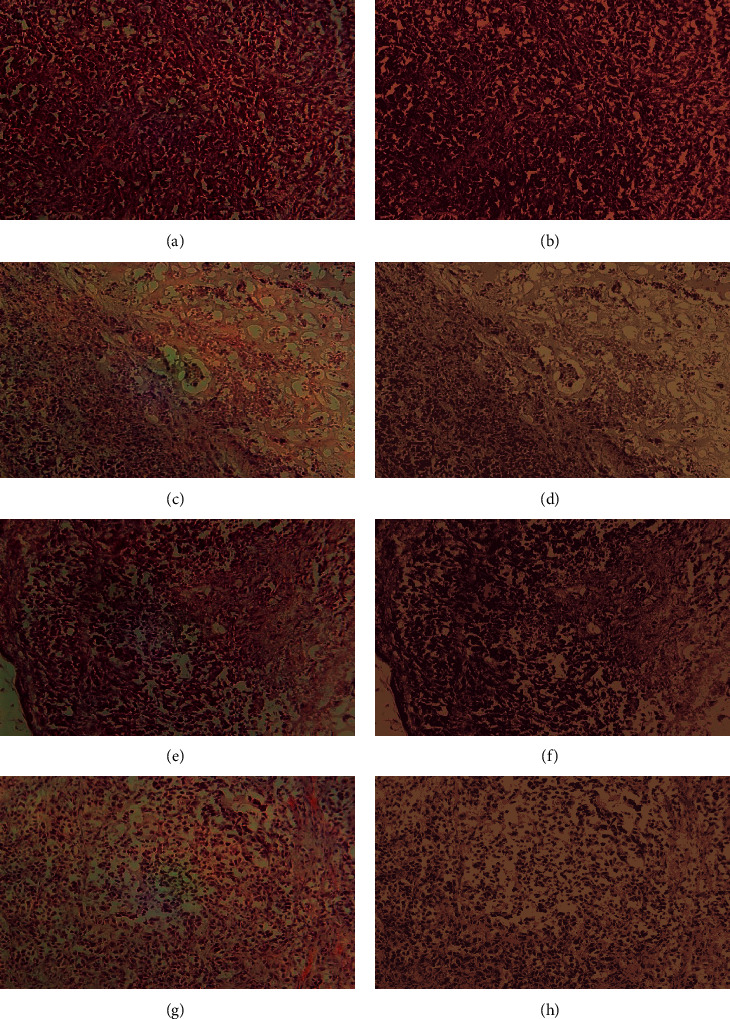
The original images of dataset 1 with their segmented images using the proposed approach: (a–d) the original images and (e–h) their segmented images.

**Figure 11:**
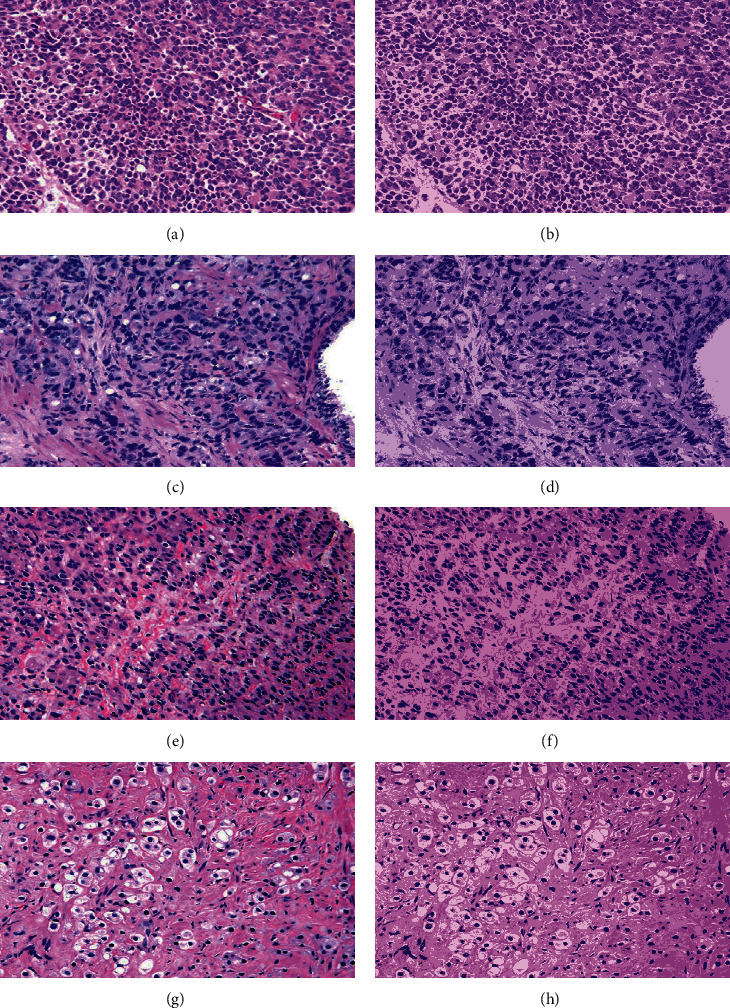
The original images of dataset 2 with their segmented images using the proposed approach: (a–d) the original images and (e–h) their segmented images.

Furthermore, Figures 8 and 9 visualize clusters' regions with their labels, for example, images taken from dataset 1 and dataset 2, respectively. It is clear to see that the method is able to cluster properly images' pixels in their appropriate regions. Figures 10 and 11 display the original images of dataset 1 and dataset 2 with their clustered images. The visual vision evaluation of segmented images shows that clustering method is beneficial for segmentation, screening, and analyzing prostate cancer disease from NIR and histological color images.

For more analyzing, Tables 1 and 2 give the distribution of clusters' pixels for segmented images of dataset 1 and dataset 2.

**Figure 12:**
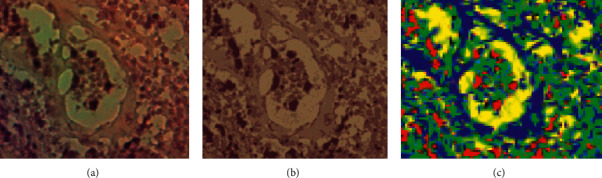
Comparison of the proposed method against the previous prostate image segmentation method: (a) part of original image 2 of dataset 1, (b) segmented image of the proposed segmentation method, and (c) segmented image of the previous segmentation method [16].

Figures 10 and 11 illustrate the resulted images of segmentation for dataset 1 and dataset 2 original images displayed in Figures 1 and 2 with a value of 4 as the predefined number of clusters, and from Tables 1 and 2 that list the number and the ratio of clusters' pixels for each segmented image, we can see that the ratios of pixels for cluster 1, cluster 2, cluster 3, and cluster 4 of image 1 in dataset 1 are 15.53%, 19.89%, 31.02%, and 33.56%. It is clear to notice that the distribution pixel ratio of cluster 1 and cluster 2 is almost near to each other and the distribution pixels ratio of cluster 3 and cluster 4 is also near to each other. Moreover, we can see that the cluster 4 that has the largest ratio is the denominator cluster. Also, we can see that the ratios of pixels for cluster 1, cluster 2, cluster 3, and cluster 4 of image 1 in dataset 2 are 22.19%, 32.48%, 25.80%, and 19.52%. The denominator cluster is cluster 2. Subsequently, we notice that there are no big differences between ratios regarding the density of clusters' distributions. Over this discussion, we compare the visual representation of segmentation results between the method developed in this work and the method proposed in previous work [16].  Figure 12 compares the segmented images of the two methods on the same original image taken from dataset 1.

As shown in Figure 12(b), it is clear that the regions of the segmented image are distinguished accurately like the regions of the original image. In Figure 12(c), the segmentation method generates some artifacts and noises regions. Accordingly, Figure 12 shows that the segmentation results of the optimized k-means-based segmentation method are interested and more appropriate for analysis than those of the previous method in terms of visual measurement. Although we obtained a high quality result, the issues of uncertain nonlinear imaging systems and unknown parameters [27, 28] may affect the segmentation results. Besides, the change in illumination and contrast of the prostate images as well as the outliers presented in the acquired input images might make it not work well. Therefore, in next work, we will apply the fuzzy c-means algorithm with elbow technique to solve such issues.

## 4. Conclusions

Reliable segmentation of prostate images is important for radiologists to analyze and detect disease. For improving visual inspection in prostate cancer diagnosis, this paper presents an optimized approach for prostate image segmentation using the k-means clustering algorithm with elbow method. The presented approach is able to solve the limitation of current methods in determining the best number of clusters for prostate image segmentation. It can segment or cluster and analyze pixels of histological and near-infrared (NIR) prostate cancer images acquired by PSMA-targeting PDT low weight molecular agents. Such agents can provide image guidance to resection of the prostate tumor and allow for the subsequent PDT to remove remaining or not eradicable cancer cells. The experimental results on two datasets of color prostate images are obtained using the optimized image segmentation approach. The analysis of segmented images with more attraction to clusters with small regions can help to attain more perfect diagnosis. Clusters' statistics and ratio results of pixels in the segmented images show the applicability of proposed approach for giving the optimum number of clusters for prostate cancer analysis and diagnosis. The proposed approach has a number of advantages such as its simplicity and its ability to solve the ambiguity that arises in the k-means algorithm to select the *k* value for prostate image segmentation. Even though there are advantages, there are some disadvantages related to the change in illumination and contrast to the prostate images as well as the outliers presented in the acquired input images. In future work, we plan to extend the proposed approach to deal with uncertainty and changes in the input prostate images, and we will combine this proposed approach with the machine learning algorithm for prostate cancer diagnosis.

## Figures and Tables

**Table 1:** The distribution of pixels in segmented images of dataset 1.

Image name	Cluster label	Number of pixels	Pixels ratio (%)
Image 1(a)	Cluster 1	75570	15.53
	Cluster 2	96800	19.89
	Cluster 3	150938	31.02
	Cluster 4	163313	33.56

Image 2(b)	Cluster 1	105475	21.22
	Cluster 2	164569	33.10
	Cluster 3	148752	29.92
	Cluster 4	78354	15.76

Image 3(c)	Cluster 1	101934	20.51
	Cluster 2	107939	21.72
	Cluster 3	134534	27.07
	Cluster 4	152537	30.70

Image 4(d)	Cluster 1	92620	29.02
	Cluster 2	106532	33.38
	Cluster 3	71996	22.56
	Cluster 4	48004	15.04

**Table 2:** The distribution of pixels in segmented images of dataset 2.

Image name	Cluster label	Number of pixels	Pixels ratio (%)
Image 1(a)	Cluster 1	38503	22.19
	Cluster 2	56362	32.48
	Cluster 3	44772	25.80
	Cluster 4	33877	19.52

Image 2(b)	Cluster 1	29566	16.95
	Cluster 2	76779	44.00
	Cluster 3	37948	21.75
	Cluster 4	30191	17.30

Image 3(c)	Cluster 1	56772	32.47
	Cluster 2	72832	41.66
	Cluster 3	22898	13.10
	Cluster 4	22344	12.78

Image 4(d)	Cluster 1	32548	18.73
	Cluster 2	75774	43.61
	Cluster 3	53014	30.51
	Cluster 4	12424	7.15

## Data Availability

The data used to support the findings of this study are available from the corresponding author upon request.
